# COI1-dependent jasmonate signalling affects growth, metabolite production and cell wall protein composition in arabidopsis

**DOI:** 10.1093/aob/mcy109

**Published:** 2018-06-19

**Authors:** Moritz Bömer, José A O’Brien, Imma Pérez-Salamó, Jovaras Krasauskas, Paul Finch, Andrea Briones, Arsalan Daudi, Puneet Souda, Tjir-Li Tsui, Julian P Whitelegge, G Paul Bolwell, Alessandra Devoto

**Affiliations:** 1 Plant Molecular Science and Centre of Systems and Synthetic Biology, School of Biological Sciences, Royal Holloway University of London, Egham, Surrey, UK; 2 Departamento de Genética Molecular y Microbiología, Departamento de Fruticultura y Enología, Pontificia Universidad Católica de Chile, Santiago, Chile; 3 Pasarow Mass Spectrometry Laboratory, Department of Psychiatry and Biobehavioral Sciences, David Geffen School of Medicine, University of California, Los Angeles, CA, USA

**Keywords:** *Arabidopsis thaliana*, cell suspension culture, COI1, jasmonate, cell cycle, cell wall proteins, primary metabolism, stress signalling

## Abstract

**Background and Aims:**

Cultured cell suspensions have been the preferred model to study the apoplast as well as to monitor metabolic and cell cycle-related changes. Previous work showed that methyl jasmonate (MeJA) inhibits leaf growth in a *CORONATINE INSENSITIVE 1* (*COI1*)-dependent manner, with COI1 being the jasmonate (JA) receptor. Here, the effect of COI1 overexpression on the growth of stably transformed arabidopsis cell cultures is described.

**Methods:**

Time-course experiments were carried out to analyse gene expression, and protein and metabolite levels.

**Key Results:**

Both MeJA treatment and the overexpression of COI1 modify growth, by altering cell proliferation and expansion. DNA content as well as transcript patterns of cell cycle and cell wall remodelling markers were altered. COI1 overexpression also increases the protein levels of OLIGOGALACTURONIDE OXIDASE 1, BETA-GLUCOSIDASE/ENDOGLUCANASES and POLYGALACTURONASE INHIBITING PROTEIN2, reinforcing the role of *COI1* in mediating defence responses and highlighting a link between cell wall loosening and growth regulation. Moreover, changes in the levels of the primary metabolites alanine, serine and succinic acid of MeJA-treated Arabidopsis cell cultures were observed. In addition, COI1 overexpression positively affects the availability of metabolites such as β-alanine, threonic acid, putrescine, glucose and myo-inositol, thereby providing a connection between JA-inhibited growth and stress responses.

**Conclusions:**

This study contributes to the understanding of the regulation of growth and the production of metabolic resources by JAs and *COI1.* This will have important implications in dissecting the complex relationships between hormonal and cell wall signalling in plants. The work also provides tools to uncover novel mechanisms co-ordinating cell division and post-mitotic cell expansion in the absence of organ developmental control.

## INTRODUCTION

Jasmonate (JA) signalling, perceived by the CORONATINE INSENSITIVE 1 (COI1) receptor (Chini *et al.*, 2007; Thines *et al.*, 2007; Fonseca *et al.*, 2009; Yan *et al.*, 2009), regulates developmental, abiotic and biotic stresses that among others, involve the cell wall (Balbi and Devoto, 2008; Howe and Jander, 2008; Gális *et al.*, 2009; Wu and Baldwin, 2010; Denness *et al.*, 2011; Noir *et al.*, 2013; Wasternack and Hause, 2013). The plant cell wall is a highly dynamic structure and an essential component involved in cell morphogenesis and plant–pathogen interactions (Cosgrove, 2005; Hématy *et al.*, 2009; Szymanski and Cosgrove, 2009). Phytohormones such as abscisic acid (ABA), salicylic acid (SA), JAs and ethylene, as well as reactive oxygen species (ROS) regulate the cross-talk between biotic and abiotic stress responses (reviewed by Fujita *et al.*, 2006; Bolwell and Daudi, 2009). JA, SA and ethylene are produced as a consequence of reduced cellulose biosynthesis associated with changes in cell wall structure and composition and increased pathogen resistance (Ellis and Turner, 2002; Cano-Delgado *et al.*, 2003; Manfield *et al.*, 2004; Hernandez-Blanco *et al.*, 2007; Hamann *et al.*, 2009). ROS- and JA-dependent processes regulate lignin biosynthesis following damage (Denness *et al.*, 2011). Plant cell cultures have been subjected to elicitation by biotic stressors and/or transiently transformed to study host defence (Kuchitsu *et al.*, 1997; Ferrando *et al.*, 2000; Day *et al.*, 2001; Navarro, 2004). O’Brien *et al.* (2012) showed, using arabidopsis cell suspension cultures, that the cell wall peroxidase genes *PRX33* and *PRX34* are required for microbe-associated molecular pattern (MAMP)-activated responses.

Cell cultures of different plants such as tobacco Bright Yellow 2 (BY-2), *Catharantus roseus* and arabidopsis have been previously subjected to treatment with methyl jasmonate (MeJA) followed by targeted metabolite analysis (Goossens *et al.*, 2003; Wolucka *et al.*, 2005; Fukusaki *et al.*, 2006; Rischer *et al.*, 2006). Extensive metabolic changes in primary and secondary metabolism were caused by MeJA treatment of *Medicago truncatula* cultures (Broeckling *et al.*, 2005). A recent study provided molecular evidence suggesting that *NtCOI1* functions upstream of the transcription factor NtMYB305 playing a role in co-ordinating plant primary carbohydrate metabolism and related physiological processes in tobacco (Wang *et al.*, 2014). For the most part, studies on metabolic profiling of arabidopsis cell cultures in response to JAs have focused on particular classes of metabolites such as monolignols (Pauwels *et al.*, 2008). Studies on the effect of MeJA on the cell cycle have also been carried out on actively dividing and synchronized cell cultures (Światek *et al.*, 2002, 2004; Pauwels *et al.*, 2008). Despite the obvious limitations due to lack of specialized organ responses, plant cell culture represents an abundant source of plant cell wall material and hence is still the system of choice to analyse related signalling.

Devoto *et al.* (2002) generated epitope-tagged COI1-overexpressing arabidopsis plants and transiently transformed cell suspensions to demonstrate that COI1 interacts with SKP1-like proteins and the histone deacetylase HDA6, forming an SCF^COI1^ complex. In this work, *Arabidopsis thaliana* cell suspension cultures have been stably transformed with *COI1*, and this system was used to analyse the effects of JA signalling on cell growth and on the production of cell wall proteins and metabolites.

Our findings frame a case study for the stable transformation of arabidopsis cell suspensions identifying *COI1*-dependent changes in cell wall proteins, cell division and expansion, as well as availability of primary metabolites. The effect of the stable overexpression of COI1 on the apoplastic proteome and on the growth dynamics of cell suspensions was analysed with the aid of flow cytometry and transcript analysis of cell cycle and cell wall remodelling markers. The results are corroborated by *in planta* studies. Changes in primary metabolism of cell suspensions were determined by gas chromatography–mass spectrometry (GC-MS) analysis identifying *COI1*- as well as MeJA-dependent metabolic changes. It is shown here that the overexpression of COI1 affects polyamine and inositol metabolism and possibly glycolysis. The possible significance of MeJA-dependent changes on the level of succinic acid, an intermediate of the Krebs cycle, as well as other primary metabolites is discussed.

## MATERIALS AND METHODS

### Plant material

Arabidopsis *coi1*-16B (AT2G39940) (Ellis and Turner, 2002), cleaned from the *pen2* mutation (Westphal *et al.*, 2008; Noir *et al.*, 2013), *A. thaliana* T_2_ lines expressing COI1 as a haemagglutinin (HA) C-terminal fusion proteins (namely COV, COI1::HA) (Devoto *et al.*, 2002) and their genetic background Col *gl1* (or Col5, Nottingham Arabidopsis Stock Centre accession N1644) were used.

### Transformation and maintenance of arabidopsis cell cultures

Arabidopsis ecotype Landsberg erecta (Ler) cell suspension cultures derived from undifferentiated calli were transformed with *Agrobacterium tumefaciens* adapting the method of Ferrando *et al.* (2000) and O’Brien *et al.* (2012), with the construct containing the intron-tagged *COI1* (Devoto *et al.*, 2002). The suspension cultures were maintained in Murashige and Skoog basal salts with minimal organics (MSMO) medium (Sigma) containing sucrose (30 g L^–1^), naphthalene acetic acid (0.5 mg L^–1^) and kinetin (0.05 mg L^–1^), and buffered to pH 5.6–5.7 with sodium hydroxide. The cultures were kept under low light intensity (80 μmol m^–2^ s^–1^) in a continuous light regime.

### Treatment of seedlings and cell cultures with methyl jasmonate

Arabidopsis seedlings (9, 13 and 19 d after stratification) were grown and treated according to Noir *et al.* (2013). The kinematic analysis of the first true leaves of Col *gl1* and COV was performed according to Noir *et al.* (2013).

Arabidopsis Ler cell cultures were treated with medium containing 50 μM MeJA or the equivalent volume of ethanol (final concentration 0.05 %) 24 h after being transferred to new medium for the treatment duration indicated.

### Molecular biology techniques

Purification of total RNA from plant material was performed using the RNeasy Plant Mini Kit (Qiagen), and cDNA was synthesized using the QuantiTect Reverse Transcription kit (Qiagen).

Quantitative real-time amplification (qRT-PCR) in the presence of SYBR Green was performed using the SYBR^®^GREEN jumpstart taq readymix (Sigma) adapting the protocol from Noir *et al.* (2013). AT5G55480 was used as a reference gene as per Noir *et al.* (2013), and the ΔΔCt (Schmittgen and Livak, 2008) method was applied for the calculations. Primers (Supplementary Data Table SI) were designed using QuantPrime (http://quantprime.mpimp-golm.mpg.de/) (Arvidsson *et al.*, 2008).

SDS–PAGE was carried out according to Laemmli (1970) in a BioRad unit. Protein staining was performed using 0.25 % Coomassie brilliant blue (Imperial Protein staining solution, Sigma). Total protein extractions were performed according to Devoto *et al.* (2002), and protein concentration was determined by the Bradford method (Protein Assay, Bio-Rad). For western blotting, 10–15 μg of total protein was loaded and analysis was performed according to Devoto *et al.* (2002). The following antibodies were used: peroxidase-coupled monoclonal anti-HA antibody 3F10 (1:1000; Roche) and COI1 antiserum (1:1000; Agrisera).

### Ploidy measurement

Ploidy levels were measured using the Cystain UV Precise P high-resolution DNA staining kit (Partec) adapting a procedure from Dolezel *et al.* (2007) and Noir *et al.* (2013). Flow cytometry experiments were repeated at least three times for each genotype using independent biological replicates.

### Arabidopsis protoplasts isolation and imaging

For cell wall digestion 3 mL of PCV (packed cell volume) was used for 0, 2, 4 and 6 days after sub-culturing (DASU). Protoplasts were isolated as previously described (Mathur *et al*., 1995) and counted using a haemocytometer (Fuchs-Rosenthal). The protoplasts were imaged using a Nikon NiE Upright microscope, and cell number and cell volume were analysed with ImageJ (http://imagej.nih.gov/ij/).

### Proteolytic digestion and identification of peptides by nano-liquid chromatography with tandem mass spectrometry

Extraction of apoplastic washing fluid (AWF) and in-gel trypsin digestion of polypeptides for mass spectrometry was performed according to O’Brien *et al.* (2012). Mass spectrometry was performed on a hybrid linear ion-trap orbitrap instrument (Orbitrap XL, Thermo Scientific) using a high-resolution precursor measurement (filtered at <10 ppm) and low-resolution product ion spectra on the ion-trap. Peptide identifications were made using Mascot software (Matrix Sciences).

### Analysis of polar metabolites by GC-MS

Four independent biological replicates for wild type and COV samples either untreated, mock treated (ethanol vehicle) or 50 μm MeJA treated (24 samples in total) were analysed. Samples for metabolite analysis by GC-MS were prepared according to Gullberg *et al.* (2004). Metabolomic analysis was performed on a Hewlett Packard 5890 Series II gas chromatograph equipped with a Hewlett Packard 7673 Autosampler and a 25 × 0.22 mm id DB5 column with 0.25 μm film, interfaced to a Hewlett Packard 5970 mass sensitive detector (Agilent Technologies, Stockport, UK). GC-MS analysis was carried out according to O’Brien *et al.* (2012). The data were analysed with Chemstation software (Agilent) and mass spectra were extracted using AMDIS 32 v.2.72 (Automated Mass Spectral Deconvolution and Identification System, http://amdis.net/index.html) and submitted to the NIST 2014 (National Institute of Science and Technology, Gaithersburg, MD, USA; http://www.nist.gov/index.html) and Golm Metabolome Database (GMD) (Hummel *et al.*, 2010) mass spectra libraries. Only the features confirmed with both databases were selected.

### Data analysis

Individual chromatogram peak areas above threshold (peak area >7000 TIC units) expressed as ratios to the total peak areas were processed. Relative metabolite abundances were tested for statistical significance using R (R Development Core Team, 2011). The multiple comparison method was a Tukey HSD test, following a two-way analysis of variance (ANOVA; the response variable was metabolite abundance and the model was treatment × cells). For all experiments described, at least three independent biological replicates were tested, unless otherwise stated. The s.e. is shown as ± of the mean. All graphs, tables and volcano plots were produced using Microsoft Office Excel 2010.

## RESULTS

### MeJA and COI1 overexpression repress cell proliferation in stably transformed arabidopsis cell cultures

**Fig. 1.**
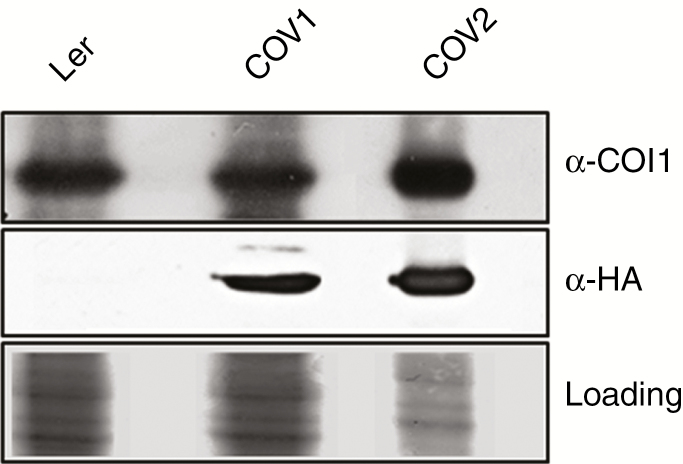
Detection of COI1::HA in Ler and COI1-overexpressing cell suspension cultures. Total protein was extracted from the wild type (Ler) and the COI1-overexpressing cell suspensions (COV1 and COV2) at 4 DASU, and run on a 12 % SDS–polyacrylamide gel. COI1 and COI::HA mass was approx. 67–68 kDa. A 10 μg aliquot of protein extract were loaded for Ler and COV1, and 2 μg for COV2.

To generate COI1-overexpressing plant cells, Ler arabidopsis cell cultures (representing the wild type and referred to here as Ler) were transformed with the *35S::COI1::HiA* construct as described before (Devoto *et al.*, 2002). Two separate microcolonies were selected to generate independent stable cell suspensions (referred to here as COV, COI-overexpressing, COV1 and COV2). Ectopic expression of the COI1::HA protein was confirmed in both COV1 and COV2 cell cultures by immunodetection using an HA-specific antibody. Higher levels of COI::HA were detected in COV2 in comparison with COV1 (Fig. 1).

**Fig. 2.**
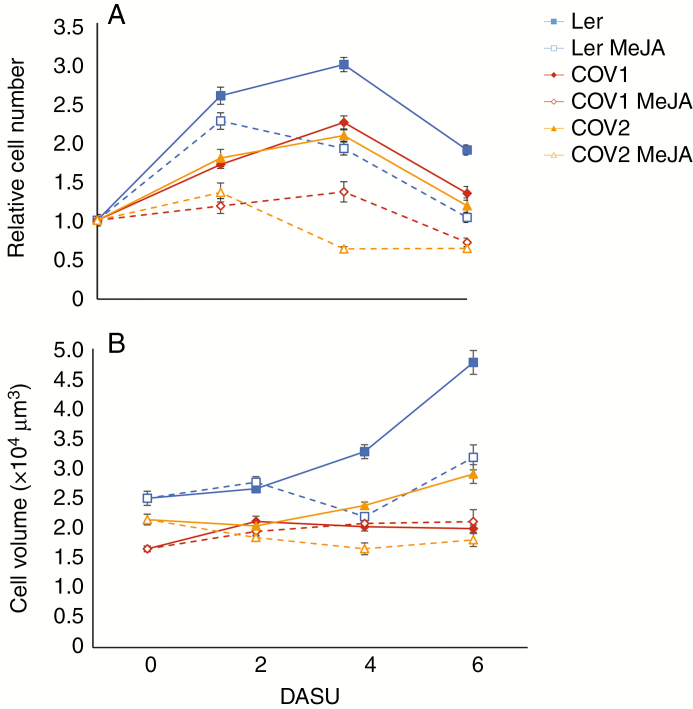
Cell number (A) and volume (B) of Ler and COV cell suspensions. Cells were treated with 50 μm MeJA at 1 DASU. MeJA-treated and untreated cell suspensions were collected at 2 d intervals and subjected to enzymatic digestion to release protoplasts, from which the cell number (cell mL^–1^) and cell volume (μm^3^) was determined using the Image J software (Schneider *et al.*, 2012). (A and B) Data represent the average of five independent biological replicates ± s.e. per line with *n* = 417–3145.

It was shown previously that MeJA affects the cell cycle via *COI1* in arabidopsis plants (Noir *et al.*, 2013). To study the effect of COI1 overexpression on cell proliferation in the absence of organ developmental control, the increase in relative cell numbers over time in Ler, COV1 and COV2 cell suspensions was compared. All three cultures exhibited higher relative cell numbers on subsequent days, with a maximum at 4 DASU (Fig. 2A). While relative cell number in Ler culture showed an approx. 3-fold increase at 4 DASU, the increase in COV1 and COV2 cultures reached about 2-fold. Following MeJA treatment (50 μm) at 1 DASU, the cell numbers decreased. At 4 DASU, the relative cell number of the Ler culture was reduced by about 36 %, whereas the JA receptor-overexpressing COV1 and COV2 cultures showed an approx. 39 % and approx. 69 % decrease, respectively (Fig. 2).

### MeJA and COI1 overexpression arrest the cell cycle in G_2_/M transition

**Fig. 3.**
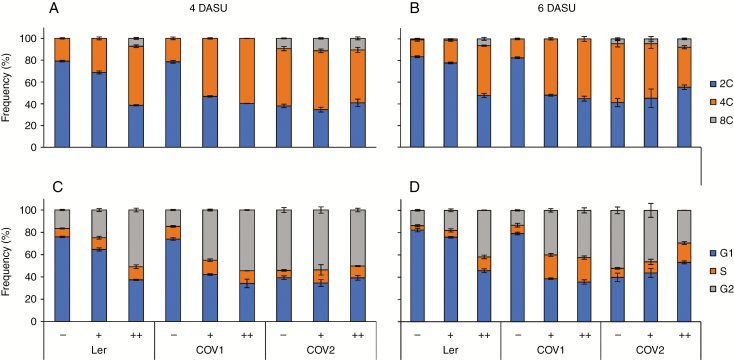
MeJA alters cell cycle progression in Ler and COV cell suspensions. Quantitative analysis of nuclear DNA content in Ler and COV cell suspensions performed by flow cytometry analysis of cell suspensions at 4 and 6 DASU treated with 50 μm (+) and 200 μm (++) MeJA at 1 DASU. (A and B) Average frequencies of the observed ploidy levels of a minimum of three independent biological replicates ± s.e. (C and D) Cell cycle analysis of flow cytometry data at 4 (C) and 6 (D) DASU. The analyses were performed on at least 20 000 nuclei isolated for each ploidy measurement.

To investigate further the effects of COI1 overexpression and MeJA elicitation on cell cycle progression, the DNA content of the cultured cells was measured by flow cytometry (Fig. 3A, B; Supplementary Data Table SII and Fig. S1). At 4 DASU, and measuring cell division parameters (Fig. 3C, D), most of the Ler cells were in G_1_ phase (approx. 76 %), and a very similar distribution was observed in COV1, with approx. 74 % of the cells in G_1_. In contrast, in COV2, a decreased frequency of G_1_ phase cells was detected (approx. 40%), with most of the cells being in G_2_/M. After MeJA treatment of Ler, a shift towards G_2_/M phase was observed in the population, and this shift could be further enhanced by increasing the MeJA concentration to 200 μm. This suggests that MeJA triggered either a G_2_ arrest or exit from the cell cycle. The fact that in the wild type, following ploidy analysis, MeJA was observed to lead to the appearance of 8C nuclei suggests that the latter had occurred in this case (Fig. 3A). In COV1 cultures, the addition of 50 μm MeJA already resulted in a higher proportion of G_2_/M cells (approx. 45 %), and this could be enhanced by 200 μm MeJA (Fig. 3C), and a similar trend was observed at 6 DASU. In contrast, upon MeJA treatment, the cell cycle phase distribution was unaffected in COV2 cells at both 4 and 6 DASU. These results extend previous findings that in arabidopsis cell suspensions MeJA has a negative effect on cell proliferation by arresting cells in the G_2_ phase (Pauwels *et al.*, 2008). Significantly here, the overexpression of COI1 enhanced the MeJA sensitivity of cells towards a G_2_ cell cycle arrest, although in COV2 cell cultures the higher COI1 expression may result in an even earlier G_2_ arrest, therefore masking the effect of MeJA.

### MeJA and COI1 overexpression differentially regulate key cell cycle marker genes

**Fig. 4.**
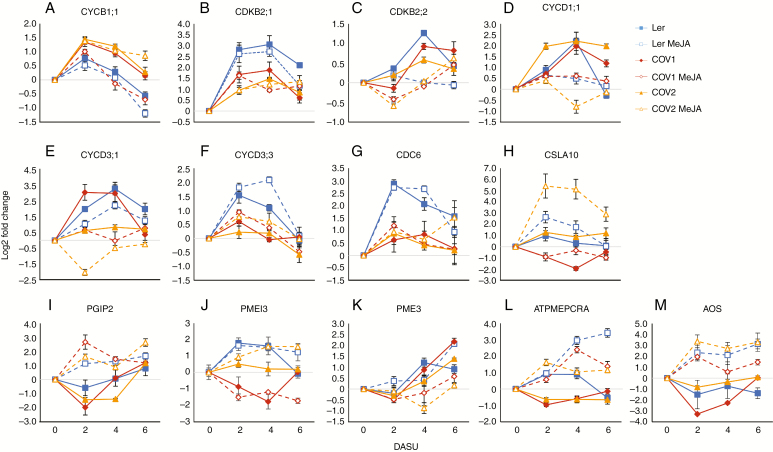
qRT-PCR analysis of cell cycle (A–G) and cell wall (H–L) remodelling markers over time. Transcript levels in Ler and COV cell suspensions by extracting RNA from 0, 2, 4 and 6 DASU cell suspensions. AT5G55480 was used as a reference gene as per Noir *et al.* (2013), and the ΔΔCt (Schmittgen and Livak, 2008) method was applied for the calculations. MeJA (50 μm) was applied to Ler, COV1 and COV2 cell suspensions at 1 DASU. The allene oxide synthase (AOS) gene was analysed to test the effectiveness of the MeJA treatment (M). Data are the averages ± s.e. of three independent biological replicates, and reactions were performed in triplicate. Results are expressed as log2 fold changes normalized to the 0 h time point for each genotype.

To gain insights into the role of COI1 overexpression in cell cycle regulation, the transcription of selected cell cycle marker genes was monitored by qRT-PCR (Fig. 4A–G). The efficacy of the MeJA treatment was assessed by analysing the expression of the *ALLENE OXIDE SYNTHASE* gene (*AOS*; AT5G42650) (Fig. 4M).

In Ler, transcription of cell cycle markers was induced for 2–4 DASU, reflecting the high mitotic activity of cells in the nutrient-rich media. As the nutrients were consumed, the activity of cell cycle markers started to decrease. Transcription of *CYCLIN B1;1* (*CYCB1;1*; AT4G37490), a checkpoint regulator at the G_2_/M transition (Dewitte *et al.*, 2003), was elevated upon sub-culturing and was the highest at 2 DASU. Throughout the culturing period, higher *CYCB1;1* levels in COV cultures were measured. MeJA treatment lowered *CYCB1;1* expression, and the rate of reduction was higher in COV1 and 2 at 4 DASU. Transcription of the G_2_/M-specific cyclin-dependent kinase genes *CYCLIN-DEPENDENT KINASE B2;1* (C*DKB2;1*; AT1G76540) and *CYCLIN-DEPENDENT KINASE B2;2* (*CDKB2;2*; AT1G20930), key regulators of cell cycle progression, was also elevated, being the highest at 4 DASU in Ler followed by COV1 and COV2. MeJA treatment decreased the expression of both *CDKB2;1* and *CDKB2;2*. *CYCD1;1* (*CYCLIN D1;1*; AT1G70210) differentially increased but peaked very similarly at 4 DASU in all three cultures. When MeJA was applied to the cell cultures, the *CYCD1;1* levels were reduced, particularly so for COV2 at 4 DASU. A known function of *CYCD3* gene products is to delay the onset of endoreduplication (Dewitte *et al.*, 2007). Expression of *CYCD3;1* (*CYCLIN D3;1*; AT4G34160) was reduced in COV2, while *CYCD3;3* (*CYCLIN D3;3*; AT3G50070) was downregulated in both COV cell cultures when compared with Ler. MeJA treatment further decreased *CYCD3;1* but not *CYCD3;3* transcript levels. In arabidopsis plants, the expression of genes required for the onset of the synthesis (S) phase, such as *CELL DIVISION CONTROL 6* (*CDC6/CDC6A*; AT2G29680), was shown to be downregulated by MeJA, consistently with the reduction of cell proliferation and the repression of endoreduplication (Noir *et al.*, 2013). In Ler cultures, the reduction in expression of this S-phase marker is associated with the overexpression of COI1 but not with MeJA treatment. Similarly, the expression levels of *CDKB2;1*, *CDKB2;2,* and *CYCD1;1* are lower in COV cultures.

To summarize, gene expression analysis demonstrated that MeJA has a negative effect on the transcription of cell cycle genes and indicated that the effects of COI1 overexpression did not always correlate with the MeJA treatments; this could suggest a MeJA-independent *COI1* function specifically related to organ developmental control.

### MeJA and COI1 overexpression reduce protoplast volume and alter the expression of genes encoding cell wall-modifying enzymes

The size of cultured cells is affected by a decrease of osmolality of the media caused by nutrient depletion (Felix *et al.*, 2000). Changes in the average protoplast volume of all three cultures over time were observed (Fig. 2B). At 6 DASU, Ler cells reached a 2-fold increase in protoplast volume compared with day 0, but COI1 overexpression prevented normal cell enlargement in both COV1 and COV2. Treatment with MeJA led to reduced cell volume in Ler cultures and resulted in only an approx. 1.3-fold increase at 6 DASU. Moreover, MeJA had no effect on COV1 cell sizes, and notably COV2 cells were more sensitive to the treatment.

MeJA negatively affects cell cycle progression during leaf development in arabidopsis (Noir *et al.*, 2013). The leaf area of *in vitro* grown Col *gl1* and of lines overexpressing COI1 (Devoto *et al.*, 2002) was measured here. The first true leaves of COV plants were analysed according to Noir *et al.* (2013) (Supplementary Data Fig. S2). Kinematic analysis confirmed that leaf growth was inhibited by MeJA treatment on average by about 80 % for both lines. Average cell area was also consistently reduced by the treatment, as was cell number, as previously demonstrated (Noir *et al.*, 2013). Here the average leaf area of untreated COV leaves is smaller than that of Col *gl1* especially during the earlier stages of leaf development (Supplementary Data Fig. S3). These observations not only are in agreement with the data obtained in cell culture but also complement our previous data showing larger leaf size for the *coi1*-16B mutant (Noir *et al.*, 2013). At the same time, increased levels of the JA receptor *in planta* do not necessarily enhance the response to the phytohormone.

As changes in cell volume also depend on cell wall elasticity (Zonia and Munnik, 2007), the expression of genes involved in cell wall remodelling was studied (Fig. 4H–L). Cellulose synthase-like A (CSLA) proteins regulate the synthesis of mannan polysaccharides, structural constituents of the cell wall (Goubet *et al.*, 2009). It was previously shown that one of the *CSLA* family genes, *CELLULOSE SYNTHASE LIKE A10* (*CSLA10;* AT1G24070), is induced by MeJA in arabidopsis (Noir *et al.*, 2013). In Ler, *CSLA10* transcription increased after sub-culturing, peaked at 2 DASU, then gradually decreased. In COV2, the *CSLA10* expression pattern was similar to that of Ler. MeJA treatment increased the transcription of *CSLA10*, particularly in COV2 cultures. The *POLYGALACTURONASE-INHIBITING PROTEIN 2* (*PGIP2*; AT5G06870) gene inhibits cell wall loosening to hinder the activity of pathogen polygalacturonases, thereby preventing cell expansion (O’Brien *et al.*, 2012). Here, *PGIP2* expression had a similar pattern in all cultures, with an initial decrease at 2 DASU followed by a linear increase. COI1 overexpression lowered *PGIP2*; however, MeJA elicitation triggered its transcription in all cultures, reaching a maximum at 2 DASU with generally higher levels in COV cultures. The inducibility of the *PGIP2* transcripts by MeJA is also consistent with previous *in planta* data (Ferrari *et al.*, 2003).

Cell wall pectins are highly methyl esterified, and their de-esterification by pectin methylesterases (PMEs) increases cell wall rigidity (Parre and Geitmann, 2005), which plays a crucial role in defence against pathogens. Moreover, pathogen-induced PME activity depends on JA signalling (Bethke *et al.*, 2014). PMEs are counteracted by methylesterase inhibitors (PMEIs) (De Caroli *et al.*, 2011); this action contributes to cell wall remodelling during growth (Lionetti *et al.*, 2012). The expression of the characterized *PECTIN METHYLESTERASE INHIBITOR 3* (*PMEI3*; AT5G20740), *PECTIN METHYLESTERASE 3* (*PME3*; AT3G14310) and *METHYLESTERASE PCR A* (*ATPMEPCRA;* AT1G11580) was tested. MeJA induced the transcription of *ATPMEPCRA*, also in agreement with data reported in Genevestigator (Hruz *et al.*, 2008). However, the expression of this gene, and of *PME3*, was downregulated by COI1 overexpression.

### COI1 overexpression induces changes in cell wall protein abundance

**Fig. 5.**
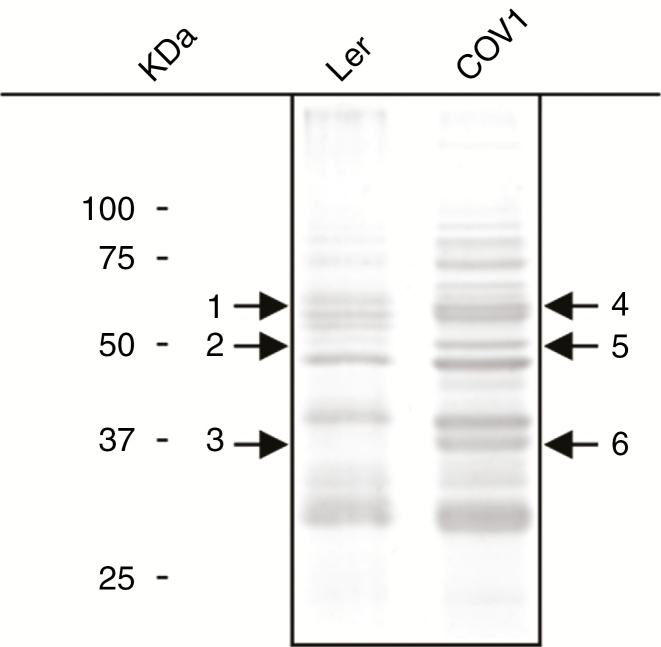
SDS–PAGE analysis of apoplastic proteins in Ler and COV cell suspensions. Ler and COV cell cultures grown for 7 d were gently vacuum filtered and then incubated for 30 min in 200 mm CaCl_2_ as described in the Materials and Methods. Proteins were precipitated with chloroform/methanol, resuspended in sample buffer and separated by SDS–PAGE. The gels were stained with Coomassie blue R250 for protein visualization. Arrows indicate bands selected for in-gel trypsin digestion and sequencing. Protein IDs are listed in Table 1 and peptides are listed in Supplementary data Table SIII. A representative SDS–polyacrylamide gel is shown of three independent experiments.

As the alteration of cell growth may be linked to cell wall-related changes, the apoplastic proteome of cell cultures overexpressing COI1 was analysed. In previous studies using the same arabidopsis Ler cell suspension culture, cytosolic contamination was deemed negligible as it was below the detection limit in CaCl_2_ extracts (Chivasa *et al.*, 2002; O’Brien *et al.*, 2012). The CaCl_2_-extracted cell wall proteins were analysed in COV1 (with protein expression levels of the COI1::HA fusion more similar to the native endogenous levels; Fig. 1) and the more abundant proteins compared with Ler were selected (Fig. 5, bands 4, 5 and 6 in COV1). No obvious protein abundance changes were detected in CaCl_2_-extracted cell wall proteins in MeJA-treated samples compared with untreated samples, in both Ler and COV1 samples (Supplementary Data Fig. S3). However, differences were identified between Ler and COV1 samples as shown in Fig. 5 (bands 1 and 4, 2 and 5, 3 and 6; Table 1). The proteins were identified as OLIGOGALACTURONIDE OXIDASE 1 (OGOX1; Benedetti *et al.*, 2018; AT4G20830; bands 1 and 4), BETA-GLUCOSIDASE (AT3G18080; bands 1 and 4)/ENDOGLUCANASES (AT1G71380/AT1G70710; bands 2 and 5) and PGIP2 (bands 3 and 6) (Supplementary Data Table SIII). The defence-related protein PGIP2 (Devoto *et al.*, 1998; Ferrari *et al.*, 2006) was visibly more abundant in COV1 compared with Ler.

**Table 1.** Proteins identified by in-gel trypsin digestion of CaCl_2_-extracted cell wall proteins of arabidopsis Ler wild type (WT) and Ler COV

Band no.	AGI number	Description	Score	Pep.#	Cover %
			Ler WT//Ler COV		
1/4	AT4G20830	Oligogalacturonide oxidase 1	1254//43	24//4	48//9
1/4	AT3G18080	Beta-glucosidase	1361//3495	72//57	55//57
2*/5*	AT1G71380/AT1G70710	Endoglucanases	5236/2345//4026/1525	10//6	33//17
3/6	AT5G06870	Polygalacturonase Inhibiting protein 2 (PGIP2)	3460//2904	12//11	38//39

Pep.#, number of peptides; Cover %, protein coverage expressed as a percentage; Score, threshold set at *P* < 0.05.

*Only the proteins with the highest scores are shown. The Score value was taken from MASCOT and represents the probability that the protein identified is not random and is based on the peptides identified.

### COI1 overexpression induces changes in whole-cell metabolites

Available studies on metabolic profiling of arabidopsis cell cultures in response to JAs are extremely diverse and do not yield a coherent picture. In this study, whole-cell extracts of Ler and COV1 cell cultures at 2 DASU were profiled by GC-MS as *O*-methyloxime-trimethylsilyl derivatives for the analysis of amino acids, monosaccharides, fatty acids and organic acids. GC-MS chromatograms showed approx. 68 peaks, representing putative metabolites (Supplementary Data Table SIV). The relative abundances of each peak were processed as a ratio of the total peak area and tested for statistical significance. The metabolites that were differentially regulated across all biological replicates were selected. A pair-wise comparison was carried out between untreated Ler and COV cells. Both lines were treated with 50 μm MeJA for 24 h and pair-wise comparisons between mock-treated and MeJA-treated cells were performed within Ler and COV.

**Fig. 6.**
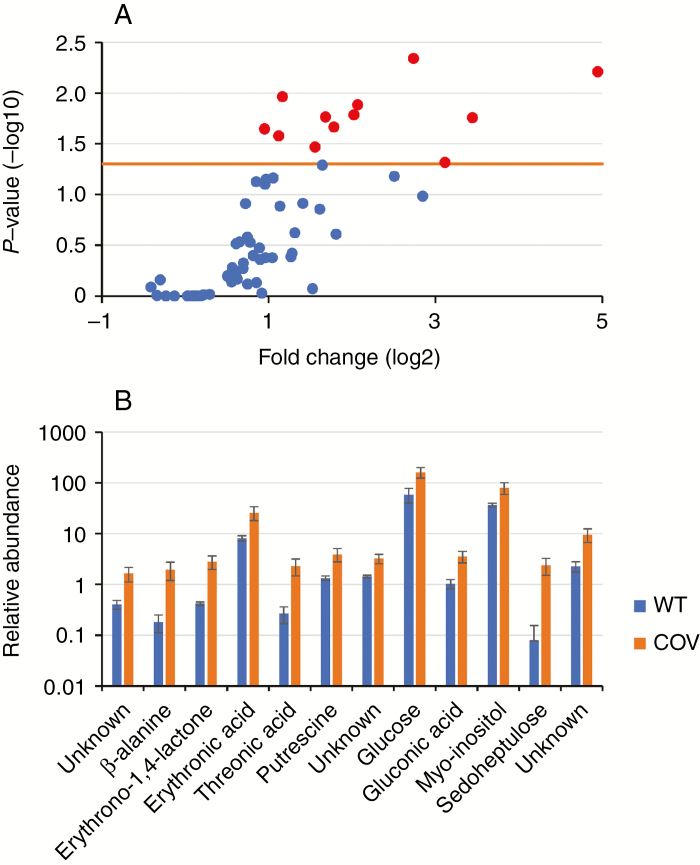
Differentially regulated metabolites in arabidopsis cell culture associated with the overexpression of the JA receptor COI1. Significant metabolite changes in COV1 cell culture compared with Ler (both untreated) using GC-MS. (A) Volcano plot of metabolomics data. The *x*-axis is the mean ratio fold change (plotted on a log2 scale) of the relative abundance of each metabolite between untreated Ler and COV1. The *y*-axis represents the statistical significance *P*-value (plotted on a –log10 scale) of the ratio of relative abundances for each metabolite. Metabolites highlighted in red (and also represented in B) hyperaccumulate in COV1 cell culture and have significant *P*-values (orange threshold bar represents *P* < 0.05) and high fold change values (>2). (B) The vertical scale bars (log10) represent the relative metabolite abundance normalized to the total peak areas. Metabolites shown are significantly different (*P* < 0.05) according to pair-wise comparison using Tukey HSD test. Data represent the means of four independent biological replicates and error bars represent the s.e.

Twelve significant metabolite changes were identified in COV when compared with Ler and are therefore associated with the overexpression of COI1 (Fig. 6). Metabolites accumulating in COV1 with significant *P*-values (*P* < 0.05) and fold change values >2 (except for glucose) are shown (Fig. 6A). Relative mean abundances of β-alanine, erythrono-1,4-lactone, erythronic acid, threonic acid, putrescine, glucose, gluconic acid, myo-inositol, sedoheptulose and two unknown metabolites are plotted (Fig. 6B), identifying glucose as the most abundant among the differentially regulated metabolites and 1.9-fold upregulated in COV1 (*P* < 0.026). Most metabolites appear to accumulate at higher levels in COV1 relative to Ler, indicating a change in primary metabolism associated with the overexpression of the COI1 receptor, but only the above-mentioned 12 metabolites pass the significance threshold (Fig. 6A). Four significant metabolite changes were identified within Ler or COV1 cell culture following MeJA treatment (Supplementary Data Fig. S4). Succinic acid was the only significantly changed metabolite found in Ler; it was 2.2-fold upregulated (*P* < 0.001) following MeJA treatment (Table 2). Succinic acid was also significantly upregulated (*P* < 0.004) in MeJA-treated COV1 samples (Table 2) together with the amino acids alanine (*P* < 0.013) and serine (*P* < 0.036) that were also shown to be significantly upregulated in COV1 upon MeJA treatment (Table 2).

**Table 2.** Differentially regulated metabolites in 2-day-old Ler and COV1 cell cultures following 50 µm MeJA treatments for 24 h analysed using GC-MS.

Metabolite	Ler	COV1	COV1/Ler		Ler				COV1			
	UN	UN	Fold	*P*	Mock	MeJA	Fold	*P*	Mock	MeJA	Fold	*P*
Unknown	0.4 ± 0.08	1.64 ± 0.526	4.07	0.016*	0.56 ± 0.107	0.37 ± 0.028	0.67	0.993	0.81 ± 0.179	0.64 ± 0.07	0.79	0.995
β-Alanine	0.18 ± 0.069	1.97 ± 0.773	10.89	0.017*	0.32 ± 0.041	0.25 ± 0.013	0.81	0.999	0.66 ± 0.203	0.95 ± 0.24	1.44	0.990
Erythrono-1,4-lactone	0.42 ± 0.032	2.81 ± 0.835	6.67	0.005*	0.71 ± 0.252	1.06 ± 0.144	1.50	0.986	1.39 ± 0.15	1.55 ± 0.322	1.12	0.999
Erythronic acid	8.11 ± 1.022	26.02 ± 7.902	3.21	0.017*	10.04 ± 1.305	8.28 ± 0.385	0.83	0.999	14.23 ± 1.03	14.39 ± 1.957	1.01	0.999
Threonic acid	0.27 ± 0.096	2.31 ± 0.844	8.66	0.048*	1.69 ± 0.566	1.62 ± 0.126	0.96	0.999	1.03 ± 0.122	1.18 ± 0.399	1.14	0.999
Putrescine	1.34 ± 0.131	3.93 ± 1.156	2.94	0.034*	1.79 ± 0.374	1.47 ± 0.356	0.82	0.998	2.34 ± 0.294	2.4 ± 0.277	1.02	0.999
Unknown	1.44 ± 0.083	3.23 ± 0.689	2.24	0.011*	1.6 ± 0.188	1.44 ± 0.071	0.90	0.999	2.17 ± 0.059	1.94 ± 0.319	0.90	0.996
Glucose	119.38 ± 5.558	230.63 ± 52.398	1.93	0.023*	114.84 ± 5.217	161.81 ± 7.307	1.41	0.664	154.07 ± 5.796	210.77 ± 4.559	1.37	0.478
Gluconic acid	1.04 ± 0.216	3.56 ± 0.897	3.44	0.022*	1.16 ± 0.107	0.82 ± 0.186	0.70	0.996	1.85 ± 0.189	2.55 ± 0.742	1.38	0.914
Myo-inositol	36.74 ± 3.011	79.97 ± 20.922	2.18	0.026*	36.82 ± 2.204	35.47 ± 1.312	0.96	0.999	46.52 ± 1.355	31.18 ± 1.944	0.67	0.812
Sedoheptulose	0.08 ± 0.077	2.39 ± 0.879	30.84	0.006*	0 ± 0	0.1 ± 0.1	N/A	0.999	1.18 ± 0.24	1.51 ± 0.259	1.28	0.990
Unknown	2.28 ± 0.517	9.53 ± 2.845	4.19	0.013*	2.69 ± 0.493	2.19 ± 0.262	0.81	0.999	5.63 ± 0.467	5.36 ± 1.365	0.95	0.999
Succinic acid	2.29 ± 0.086	2.09 ± 0.541	0.91	0.998	2.81 ± 0.079	6.15 ± 0.243	2.19	0.000^†^	1.04 ± 0.075	3.1 ± 0.54	2.99	0.004^†^
Alanine	1.33 ± 0.145	2.48 ± 0.841	1.86	0.439	2.76 ± 0.164	3.74 ± 0.125	1.35	0.602	1.41 ± 0.142	3.75 ± 0.562	2.66	0.013^†^
Serine	1.21 ± 0.159	2.5 ± 0.935	2.06	0.419	2.5 ± 0.146	3.71 ± 0.075	1.49	0.476	1.61 ± 0.238	3.84 ± 0.594	2.39	0.036^†^

Differences in relative mean abundances ± s.e and fold changes are presented.

*P < 0.05 of COV1 compared with Ler, both untreated (UN).

^†^ *P* <0.05 comparing MeJA and mock treatment in Ler wild type or Ler COV samples.

## DISCUSSION

### A role for COI1 in regulating the cell cycle and cell wall remodelling

Stably transformed arabidopsis Ler cell cultures (COV) have proven to be a reliable system to study the growth dynamics of plant cells, and to analyse related gene and protein expression and changes in metabolism. Our results showed that MeJA treatment and COI1 overexpression negatively affected growth in cell cultures. A degree of synergy between the levels of COI1 overexpression and MeJA treatment on the repression of cell proliferation was observed (Figs 1 and 2).

Jasmonate-mediated metabolite production has been used for pharmaceutical and biotechnological purposes (Patil *et al.*, 2014), and the accumulation of metabolites in response to MeJA elicitation reduces cell growth in *Nicotiana tabacum* BY-2 and *Panax ginseng* cell suspensions (Goossens *et al.*, 2003; Thanh *et al.*, 2005). The activation of defence responses redirects energy resources from primary metabolism at the expense of growth (Patil *et al.*, 2014). Recently, the JASMONATE ZIM-DOMAIN (JAZ)–MYC transcriptional module was highlighted as the molecular basis behind the regulation of growth–defence balance (Major *et al.*, 2017*b*), pointing towards a complex signalling hub mediating cross-talk between hormone and light signalling pathways (Major *et al.*, 2017*a*). Major *et al.* (2017*a*) proposed a model suggesting that growth and defence trade-offs are a consequence of a transcriptional network that evolved to maximize plant fitness, rather than just being the result of metabolic constraints. Taken together, our findings indicate that MeJA inhibits cell growth through the JA receptor COI1, in line with the notion that JAs contribute to regulating the trade-off between defence mode and plant growth (Yang *et al.*, 2012; Noir *et al.*, 2013).

Cell cycle analysis by flow cytometry at 4 DASU, which coincides with the highest cell number in the untreated cultures, showed that MeJA promotes a shift from G_1_ to G_2_/M in Ler cells in a dose-dependent manner, suggesting a G_2_ arrest that leads to reduced or delayed cell division (Figs 1 and 3). COI1 overexpression mimics the effect of MeJA treatment which further enhanced the shift from G_1_ to G_2_/M in COV1 lines but not in COV2. MeJA was shown to block both G_1_ and G_2_/M transitions in tobacco BY2 cell cultures (Światek *et al.*, 2002) and to mediate the arrest in G_2_ phase in arabidopsis cell cultures (Pauwels *et al.*, 2008). In asynchronously dividing *Taxus* cell cultures, MeJA affected cell cycle progression by transiently increasing cells in G_2_ phase (Patil *et al.*, 2014). A role for MeJA in the regulation of cell cycle progression in arabidopsis plants was reported (Noir *et al.*, 2013), demonstrating that MeJA inhibits mitosis, arresting the cell cycle in G_1_ prior to the transition to S phase, in a *COI1*-dependent manner.

It is shown here that COI1 overexpression inhibits or delays progression of the cell cycle in cell suspensions, specifically blocking G_2_/M transition, suggesting a mechanistic difference and further clarifying the role of the JA receptor in the absence of organ developmental control.

Analysis of selected cell cycle marker genes suggested that MeJA also promotes a switch to endoreduplication in a *COI1*-dependent manner *in planta* (Noir *et al.*, 2013). However, this process is not evident in cell cultures. The accumulation of *CYCB1;1*, *CDKB2;1* and *CDKB2;2* was reduced following MeJA treatment (Fig. 4). Consistently, COI1 overexpression reduced mRNA levels of CDKBs in COV cell cultures, which further supports a role for *COI1* in mediating cell cycle progression. However, the expression of *CYCB1;1* was upregulated in COV lines in comparison with Ler cultures. This observation, counterintuitive at face value, is nevertheless in line with a previous report showing G_2_ arrest of arabidopsis root cells and *CYCB1;1* accumulation after γ-irradiation (Ricaud *et al.*, 2007).

*CYCD1;1* was shown to regulate the cell cycle positively through its function at G_0_/G_1_/S and in S/G_2_ transitions and to accelerate cell proliferation in BY-2 cells upon overexpression (Koroleva *et al.*, 2004). Here, transcripts of *CYCD1;1* were reduced upon MeJA treatment in all cell cultures, indicating that this D-type cyclin could mediate the inhibition of cell proliferation following MeJA signalling, in agreement with *in planta* data from Noir *et al.* (2013). The levels of *CYCD3;1* transcripts were reduced in COV2 and by MeJA treatment. *CYCD3;3* activity was repressed in both COV cell lines, consistent with the findings of Oakenfull *et al.* (2002). CYCD3s negatively regulate endoreduplication by extending the competency to enter mitosis (Dewitte *et al.*, 2007). The shift from G_1_/S to G_2_/M phases identified by flow cytometry could therefore be mediated by CYCD3 in a context where the endocycle is not started. The repression of *CDC6* expression in COV cultures suggests that COI1 may act on this key limiting factor to stall the S phase prior to replication and, consequently, to the G_2_ phase. The arrest of cell cycle in G_2_/M transition observed in COV (Fig. 3) could also depend on reduced COI1-dependent CDKB2 activity (Zhiponova *et al.*, 2006)

MeJA treatment and COI1 overexpression reduced the cell volume (Fig. 1B). The composition of the cell wall plays a key role in maintaining the equilibrium between osmotic pressure and cell expansion (Parre and Geitmann, 2005; Sarkar *et al.*, 2009; Pauly and Keegstra, 2016). It can be hypothesized that the MeJA/*COI1* pathway affected cell wall structure, leading to an increased cell wall rigidity hindering cell enlargement. MeJA was shown to induce the expression of the lignin precursor monolignol biosynthetic genes in arabidopsis cell cultures (Pauwels *et al.*, 2008). Moreover, JAs regulate cell wall composition as part of JA-mediated defence responses in potato, by targeting the activity of PMEs (Taurino *et al.*, 2014).

To better understand how the MeJA/*COI1* pathway regulates cell wall remodelling, the expression of genes associated with this process and JA signalling was analysed (Fig. 4). Notably, the expression of *CSLA10* was induced by COI1 overexpression in combination with MeJA treatment, ascribing a role for *COI1* in regulating production of hemicelluloses. The data are also in line with the role of JAs in regulating cellulose biosynthesis (Ellis and Turner, 2001).

During necrotrophic infection, cell wall integrity is protected by inhibitors of pathogenic cell wall-degrading enzymes (Bellincampi *et al.*, 2014). PGIP2 contributes to cell wall rigidity independently of biotic stress (O’Brien *et al.*, 2012). The expression of *PGIP2* was shown to be upregulated by MeJA, and it was undetectable in *coi1* and *jar1* mutants after *Botrytis cinerea* infection (Ferrari *et al.*, 2003). Consistently, our results showed that MeJA induced *PGIP2* transcription, and this could be further enhanced when COI1 was overexpressed.

The PMEs and their inhibitory PMEIs are also involved in cell wall remodelling (Lionetti *et al.*, 2012). PMEs remove methyl esters from the pectin polymers, and the free pectins cross-link with Ca^2+^, increasing cell wall firmness (Willats *et al.*, 2001). The activity of plant PMEs is inhibited by PMEIs during plant growth and during pathogen defence (Parre and Geitmann, 2005; Lionetti *et al.*, 2007). The induction of PME activity is dependent on JA signalling in arabidopsis and potato (Bethke *et al.*, 2014; Taurino *et al.*, 2014). A link between JA and transcription of PMEIs was also established in arabidopsis, as exogenous MeJA and ethylene could activate the pepper *CaPMEI1* promoter (An *et al.*, 2009). Overexpression of arabidopsis *PMEI1* and *PMEI2* reduces PME activity, and increases the levels of pectin methyl esterification (Lionetti *et al.*, 2007) along with root length. The overexpression of *PMEI2* also increased plant growth and the vegetative biomass yield in arabidopsis, suggesting a role in enhancing cell expansion (Lionetti *et al.*, 2010).

The reduction in transcription of *PMEI3*, *PME3* and *ATPMEPCRA* in COV cell cultures demonstrates that *COI1* regulates pectin de-esterification/methyl esterification through the selective modulation of these enzymes. The induction of *ATPMEPCRA* by MeJA suggests that the MeJA/*COI1* pathways may control cell volume by increasing de-esterification and, as a result, rigidity.

Overall, a set of genes has been identified that function as targets of the MeJA/*COI1*-dependent pathway and whose function in cell wall remodelling could justify the inhibition of cell enlargement observed in Ler and COV cells.

### Differentially regulated apoplastic proteins with a role in growth and defence

Four proteins were more abundant in the cell wall fraction of untreated COV1 cell suspensions (Fig. 5; Table 1; Supplementary Data Table SIII). These proteins have previously been identified in the cell wall (Bayer *et al.*, 2006; O’Brien *et al.*, 2012) and COI1 dependency was demonstrated in independent studies. Oligogalacturonide oxidase 1 (OGOX1) was recently characterized and specifically oxidizes oligogalacturonides. Plants overexpressing OGOX1 were also shown to improve resistance to *Botrytis cinerea* (Benedetti *et al.*, 2018). A putative *BERBERINE BRIDGE ENZYME* gene (AT2G34810) was previously found to be induced by MeJA treatment or wounding and to be *COI1* dependent (Devoto *et al.*, 2005). Consistently, the phytotoxin coronatine induced accumulation of the elicitor-responsive transcript for the berberine bridge enzyme of *Eschscholtzia californica* (Weiler *et al.*, 1994). Several glucanases also possess antimicrobial properties (Xu *et al.*, 1994; Glazebrook *et al.*, 2003). In arabidopsis, the *BETA-1,3-GLUCANASE 2* (AT3G57260) transcripts were shown to be induced by wounding and MeJA (Devoto *et al.*, 2005). Among the endoglucanases identified was Endoglucanase 9. This protein is a member of the glycosyl hydrolase family 9 (GH9) (also named endo-1,4-β-glucanase 9 or cellulase 3; AtCEL3; Urbanowicz *et al.*, 2007). In rice, the gene encoding an *ENDO-(1,3;1,4)-β-GLUCANASE* has been described to respond to wounding and MeJA, whereby it was speculated that this response induces cell wall loosening during cell elongation and expansion as a step to regenerate injured cell walls in wounded leaf tissues (Akiyama *et al.*, 2009). JAs play a role during cell wall synthesis (Koda, 1997; Cano-Delgado *et al.*, 2000; Ellis and Turner, 2001, 2002); however, the association between JAs and cell expansion is so far limited (Brioudes *et al.*, 2009). Noir *et al.* (2013) investigated the expression of genes with a role in cell expansion, revealing a complex picture made up of genes differentially regulated and *COI1* dependent during development. The interaction of AtCEL3 with cyclins in arabidopsis cell suspensions (Van Leene *et al.*, 2010) shed light on the mechanism of JA-dependent cell wall loosening to regulate cell growth. PGIPs have been shown to play a vital role in defence as extracellular inhibitors of fungal endopolygalacturonases (PGs) (Devoto *et al*., 1997, 1998; De Lorenzo *et al.*, 2001; De Lorenzo and Ferrari, 2002; Ndimba *et al.*, 2003; D’Ovidio *et al.*, 2004). In this study, the PGIP2 protein levels were more abundant in COV cell wall fractions, while *PGIP2* transcripts are induced by MeJA as previously shown (Ferrari *et al.*, 2003) (Supplementary Data Fig. S3). It is conceivable to attribute such differences to differential stability in actively dividing cells of *PGIP2* transcripts and protein levels, resulting in undetectable differences in total protein extracts following MeJA treatment.

### COI1 overexpression and MeJA treatment affect primary metabolism

The comparison of metabolic fingerprints by GC-MS in previous studies of arabidopsis leaves with those of cultured arabidopsis cells (T87 line) (Axelos *et al.*, 1992) showed similarities in the primary metabolite profiles and revealed moderate quantitative differences (Fukusaki *et al.*, 2006). Nuclear magnetic resonance (NMR) spectroscopy revealed that MeJA treatment of Arabidopsis plants increases flavonoids, fumaric acid, sinapoyl malate, sinigrin, tryptophan, valine, threonine and alanine, and decreases malic acid, feruloyl malate, glutamine and carbohydrates (Hendrawati *et al.*, 2006). A study in tobacco showed that starch metabolic genes are differentially regulated in plant tissues by *NtCOI1*, highlighting the role of the JA signalling pathway in co-ordinating plant primary metabolism (Wang *et al.*, 2014).

In this study, changes in the primary metabolism of arabidopsis Ler cell suspensions overexpressing the JA receptor COI1, as well as metabolite changes upon exposure to MeJA, were identified (Table 2). COI1 overexpression causes the accumulation of threonic acid, a product of ascorbic acid catabolism (Debolt *et al.*, 2007). Endogenous JAs may regulate steady-state ascorbic acid levels (Suza *et al.*, 2010). It is plausible that this turnover is accelerated in the transgenic cell suspension line. The increased abundance of β-alanine in COV1 cells may not be dependent on JAs in such a system, whilst MeJA increases its levels in plants (Broeckling *et al.*, 2005; Kim *et al.*, 2013).

Methyl jasmonate-triggered increases of the key amino acids alanine and serine were previously detected in *N. tabacum* (Hanik *et al.*, 2010) and *M. truncatula* (Broeckling *et al.*, 2005), providing substrates for the induction of downstream secondary metabolism for the plant defence response. In this study, levels of both amino acids were significantly increased in COV1 cells upon MeJA elicitation and appear to be more abundant in MeJA-treated Ler cells, however not significantly so.

Treatment of Ler and COV1 cells with MeJA induces succinic acid, a component of the citric acid cycle (Krebs cycle). This was previously observed in *Agastache rugosa* (Kim *et al.*, 2013) and in *M. truncatula* (Broeckling *et al.*, 2005). Such induction may be indicative of reduced turnover, while metabolic activity is rescheduled from growth to stress responses. Similarly, succinate and citric acid levels decreased in lipoxygenase (LOX)-silenced tomato fruits (Kausch *et al.*, 2012).

Glucose is an obligatory substrate of energy-producing glycolysis and polyhydroxy acids, and, together with erythronic acid, gluconic acid and threonic acid, was increased in COV1 cells. Interestingly, a study undertaken in arabidopsis showed that erythronic acid, gluconic acid and threonic acid levels increased in plants overexpressing *GLYOXALASE2-1* (*GLX2-1*) under threonine stress (Devanathan *et al.*, 2014). Whether the increase of such compounds in our study is a consequence of reduced turnover in COV1 cells rescheduling from growth to defence, and therefore directly linked with reduced growth rates in the COV1 cells, remains to be demonstrated.

Putrescine is the obligate precursor of spermidine and spermine, the major polyamines in plants. Polyamines regulate several cellular processes such as cell growth and stress tolerance (Capell *et al.*, 2004; Kasukabe *et al.*, 2004; Kusano *et al.*, 2008). In this study, putrescine levels are significantly increased in COV1 cells. Increased putrescine levels have been proposed to play a role in response to abiotic stress and wounding (Bouchereau *et al.*, 1999; Perez-Amador *et al.*, 2002; Capell *et al.*, 2004; Cuevas *et al.*, 2008). The results are also in line with studies attributing a role to JAs and *COI1* in regulating enzymes for the accumulation of putrescine (Perez-Amador *et al.*, 2002; Goda *et al.*, 2008).

Myo-inositol abundance was also increased in cells overexpressing COI1. Interestingly, InsP5 was described as a cofactor in the binding of JA-Ile to the receptor COI1, potentiating the strength of COI1–JAZ interactions (Sheard *et al.*, 2010). While a direct connection is yet to be established, our data indicate an effect of COI1 on inositol metabolism.

**Fig. 7.**
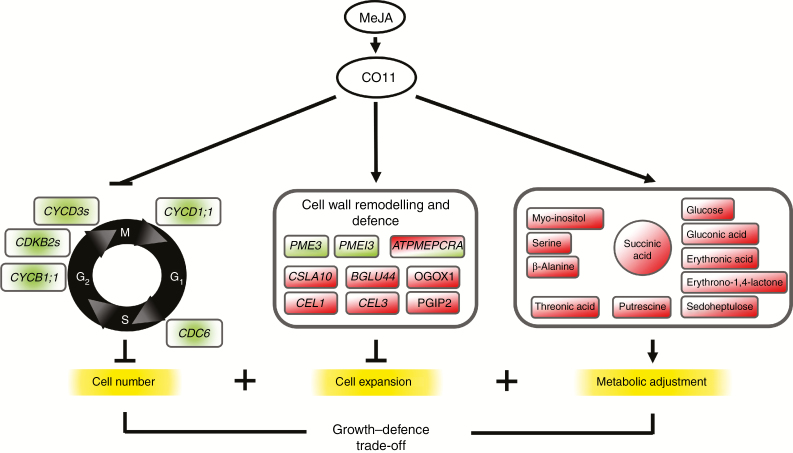
MeJA contributes to the regulation of the trade-off between defence mode and plant growth. Schematic representation of the cellular processes regulated by MeJA through the JA receptor COI1. MeJA inhibits cell proliferation via regulation of key components of the plant cell cycle and promotes changes in cell wall composition. Such modifications halt cell expansion while enhancing defence responses. MeJA induces metabolic reprogramming in plant cells to adjust to stress conditions, compromising growth. Shaded red and green shapes indicate accumulation or reduction in transcript, protein or metabolite levels, respectively.

Taken together with the finding that MeJA inhibits plant growth by repressing cell proliferation (Noir *et al.*, 2013), this study contributes to the understanding of JA- and *COI1*-mediated growth control in the context of the production of metabolic resources and the trade-off with defence responses. This knowledge will positively impact our understanding of the complex single-cell relationships between micro-organisms and plants, and their regulation by hormonal and cell wall signalling. This work also provides tools to uncover novel mechanisms co-ordinating cell division and post-mitotic cell expansion in the absence of organ developmental control. An integrated picture of the results obtained is shown in Fig. 7.

## SUPPLEMENTARY DATA

Supplementary data are available online at https://academic.oup.com/aob and consist of the following. Figure S1: flow cytometry of cell suspensions. Figure S2: COI1 overexpression *in planta*. Figure S3: SDS–PAGE analysis of cell wall proteins. Figure S4: differentially regulated metabolites in Ler and COV1. Table SI: primers used. Table SII: frequency of nuclei exhibiting 2C, 4C or 8C DNA content. Table SIII: list of peptides identified. Table SIV: list of metabolites identified.

Supplementary Data Figures S1-S4 and Tables I-II

Supplementary Data Tables III-IV
