# Supplementary material for: COI1-dependent jasmonate signalling affects growth, metabolite production and cell wall protein composition in arabidopsis
Source: Ann Bot. 2018 Jun 19;122(7):1117–29. doi: 10.1093/aob/mcy109 (PMC6324744; doi:10.1093/aob/mcy109)
Supplement: Supplementary Data Figures S1-S4 and Tables I-II [file mcy109_suppl_supplementary_figures-tables.docx]

**Title: The jasmonate receptor COI1 affects growth, metabolites production and cell wall protein composition in Arabidopsis**

Moritz Bömer^#,1,4^, Jose’ A. O’Brien^#,2^, Imma Perez-Salamo^#,1^, Jovaras Krasauskas^1^, Paul Finch^1^, Andrea Briones^1,6^, Arsalan Daudi^1,5^, Puneet Souda^2^, Tjir-Li Tsui^1^, Julian P. Whitelegge^3^, G. Paul Bolwell^1^, Alessandra Devoto^1,*^

**Supplementary data**

**Supplementary Figure Legends**

**Figure S1. Flow cytometry of cell suspensions.** Flow cytometric DNA-histograms of Ler and COV cell suspensions treated with MeJA. A-C Ler (A) untreated, (B) 50 µM MeJA, (C) 200 µM MeJA; D-F COV1 (D) untreated (E) 50 µM MeJA and (F) 200 µM MeJA; G-I COV2 (G) untreated, (H) 50 µM MeJA and (I) 200 µM MeJA. MeJA was added after 1 DASU. Representative flow cytometry peaks shown; n=>20,000 cells/sample.

**Figure S2. COI1 overexpression *in planta* affects leaf growth.** Kinematic analysis of leaf growth of Col *gl1*, and COV during development. Plants were grown in vitro in absence (-) or in presence of 50 µM MeJA (+). Leaves 1 and 2 were harvested at 9, 13, and 19 DAS to measure the leaf area (A) and the epidermal cell area (B). Values denote averages ± SE of three biological replicates (*n*=4-7) where n is the number of plants analysed per biological replicate). Panels A, B: continuous and dashed lines indicate MeJA-untreated (-) and treated (+) samples, respectively. Panel C, log10 scale. Data are representative of two independent transgenic lines. The total leaf cell number was calculated as the ratio between the average leaf area and cell area.

**Figure S3. SDS-PAGE analysis of CaCl_2_-extracted cell wall proteins comparing Ler and COV1.** Ler and COV cell cultures grown for 7 days were gently vacuum filtered and then incubated for 30 min in 200 mM CaCl2 as described in the Methods. Proteins were precipitated with chloroform/methanol, resuspended in sample buffer and separated on SDS-PAGE gel. The gels were stained with Coomassie blue R250 for protein visualization. Arrows indicate bands selected for in-gel trypsin digestion and sequencing. Protein IDs are listed in Table 1 and peptide lists in Supplemental Table III. A representative SDS-PAGE gel is shown of 3 independent experiments.

**Figure S4. Differentially-regulated metabolites in Arabidopsis Ler and COV1.** Significant metabolite changes in Ler and COV1 cell culture following 50 µM MeJA treatment for 24h. Volcano plots of metabolomics data show significant metabolite changes identified in Ler (A) and COV1 (B). The x-axis is the mean ratio fold-change (plotted on a log2 scale) of the relative abundance of each metabolite between mock and MeJA treatment. The y-axis represents the statistical significance p-value (plotted on a -log10 scale) of the ratio relative abundances for each metabolite. Metabolites highlighted in red hyper-accumulate following MeJA treatment and have significant p-values (orange threshold bar represents P < 0.05) and high fold-change values (> 2).

**Figure S1. Flow cytometry of MeJA and control cell suspensions**

**Figure S2. COI1 over-expression *in planta* affects leaf growth**

**Figure S3. SDS-PAGE analysis of CaCl_2_-extracted cell wall proteins comparing Ler and COV cell cultures**

**Figure S4. Differentially-regulated metabolites in Arabidopsis Ler and COV1 cell culture associated with MeJA treatment**

**Supplementary Tables**

**For Supplementary data Tables SIII and SIV see separate excel file.**
